# 

**Published:** 1841-06

**Authors:** 


### CONTENTS

OF NO. VI.

---

### ORIGINAL COMMUNICATIONS.

### ESSAYS AND CASES.

Art. I.—Case of Axillary Aneurism for which the Subclavian Artery was Tied; with an Inquiry into the Nature and History of that Affection. By S. D. Gross, M. D., Professor of Surgery in the Louisville Medical Institute. 401

### REVIEWS.

Art. II.—Researches on the Pathology and Treatment of some of the most important Diseases of Women: by Robert Lee, M. D., F. R. S.. Physician-Accoucheur to the British Lying-in Hospital and the Saint Mary-le-Bone Infirmary; Lecturer on Midwifery in the School of Webb street; London, 1833. p. 220. 453

### SELECTIONS FROM AMERICAN AND FOREIGN JOURNALS.

The Case of the late William Henry Harrison, President of the United States. Reported by Thomas Miller, M. D., &c. Washington City. - - - - - - - 469

### ORIGINAL INTELLIGENCE.

Medical Society of Tennessee. - - - - - 478
Death of Dr. William P. Dewees. - - - - 479
The Spring. - - - - - - - 478
Movements of Medical Men. - - - - - 478
Prize Essay. - - - - - - - 480
Our Undertaking. - - - - - - 477

